# Supplementary material for: Associations of Serum Magnesium with Brain Morphology and Subclinical Cerebrovascular Disease: The Atherosclerosis Risk in Communities-Neurocognitive Study
Source: Nutrients. 2021 Dec 16;13(12):4496. doi: 10.3390/nu13124496 (PMC8703422; doi:10.3390/nu13124496)
Supplement: Supplementary file 1 [file nutrients-13-04496-s001.zip › nutrients-1481453-SI.pdf]

Supplemental Figure S1. Forest plot of the association of magnesium with brain volumes in participants at visit 5.

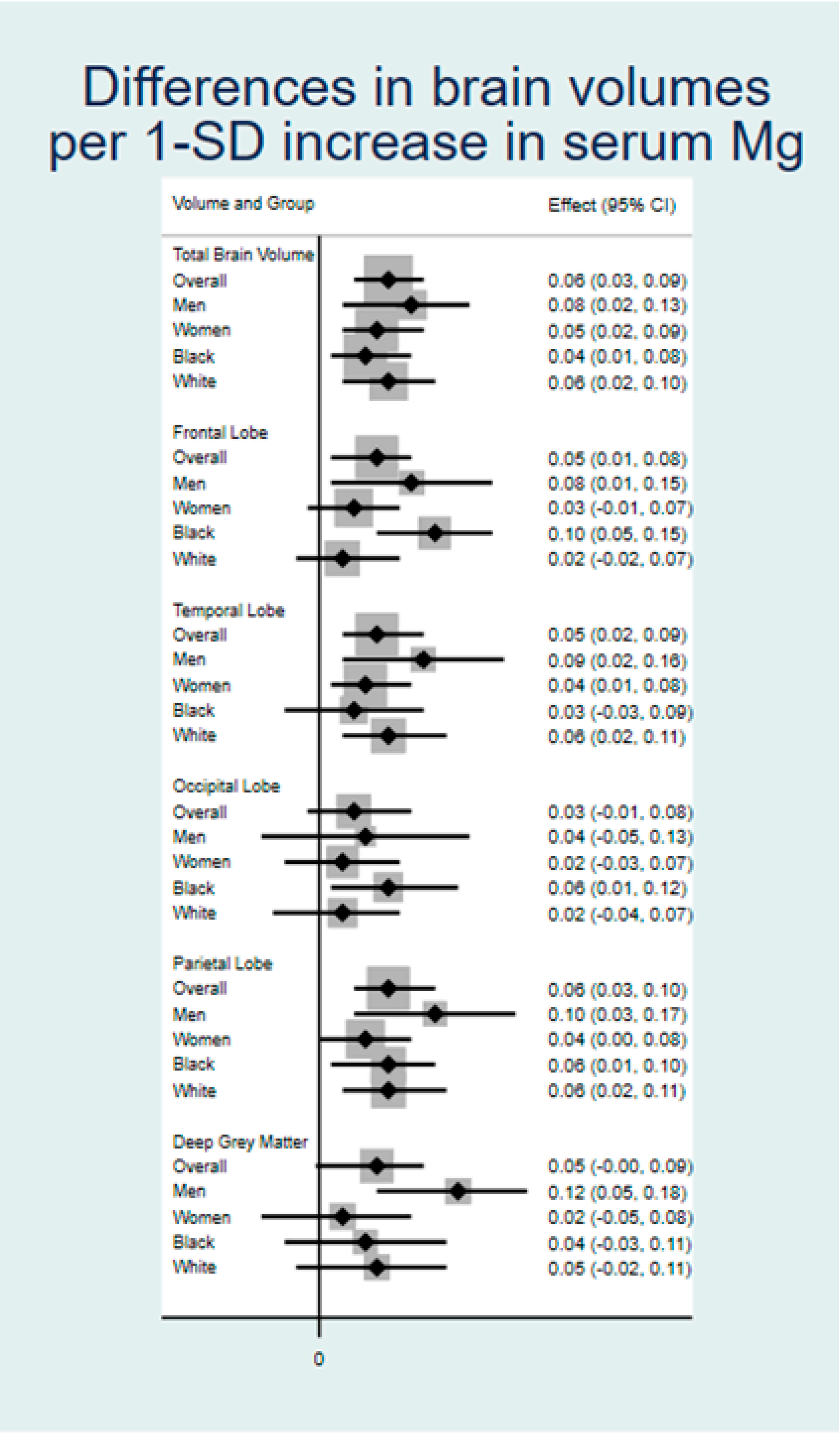

*Multiple linear regression models adjusted for age, sex, race/center, education, total intracranial volume, LDL and HDL cholesterol, body-mass index, sodium, potassium, calcium, smoking status, hypertension, hypertension medication use, history of coronary heart disease and heart failure, diabetes, eGFR, c-reactive protein, APOE allele. 1-SD Mg: 0.2 mg/dL.

Supplemental Figure S2. Forest plot of the association of magnesium with subclinical cerebrovascular disease in participants at visit 5.

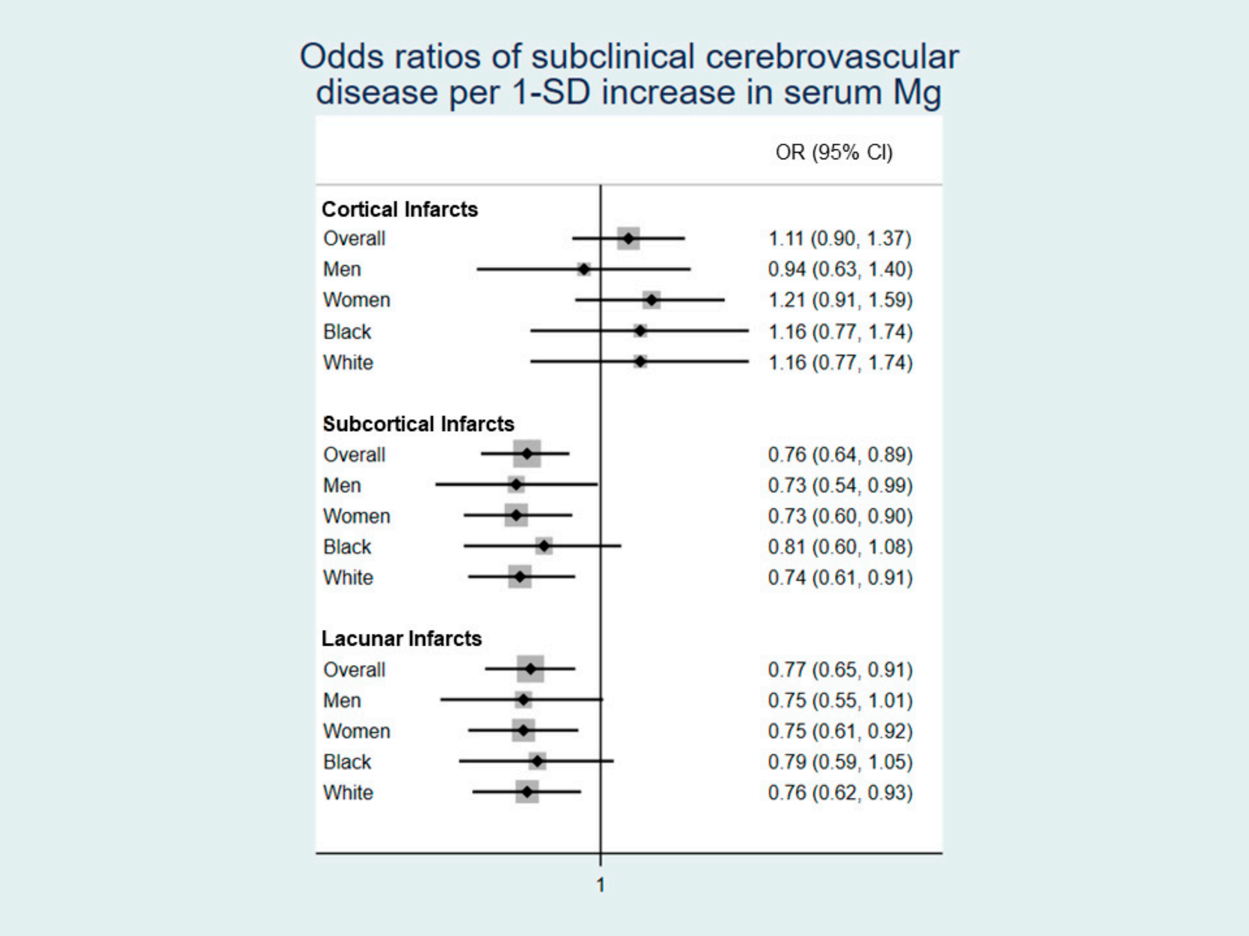

*Logistic regression models adjusted for age, sex, race/center, education, LDL and HDL cholesterol, body-mass index, sodium, potassium, calcium, smoking status, hypertension, hypertension medication use, history of coronary heart disease and heart failure, diabetes, eGFR, c-reactive protein, APOE allele. 1-SD Mg: 0.2 mg/dL.

Supplemental Figure S3. Forest plot of the association of magnesium with log-transformed white matter hyperintensity (WMH) volume in participants at visit 5.

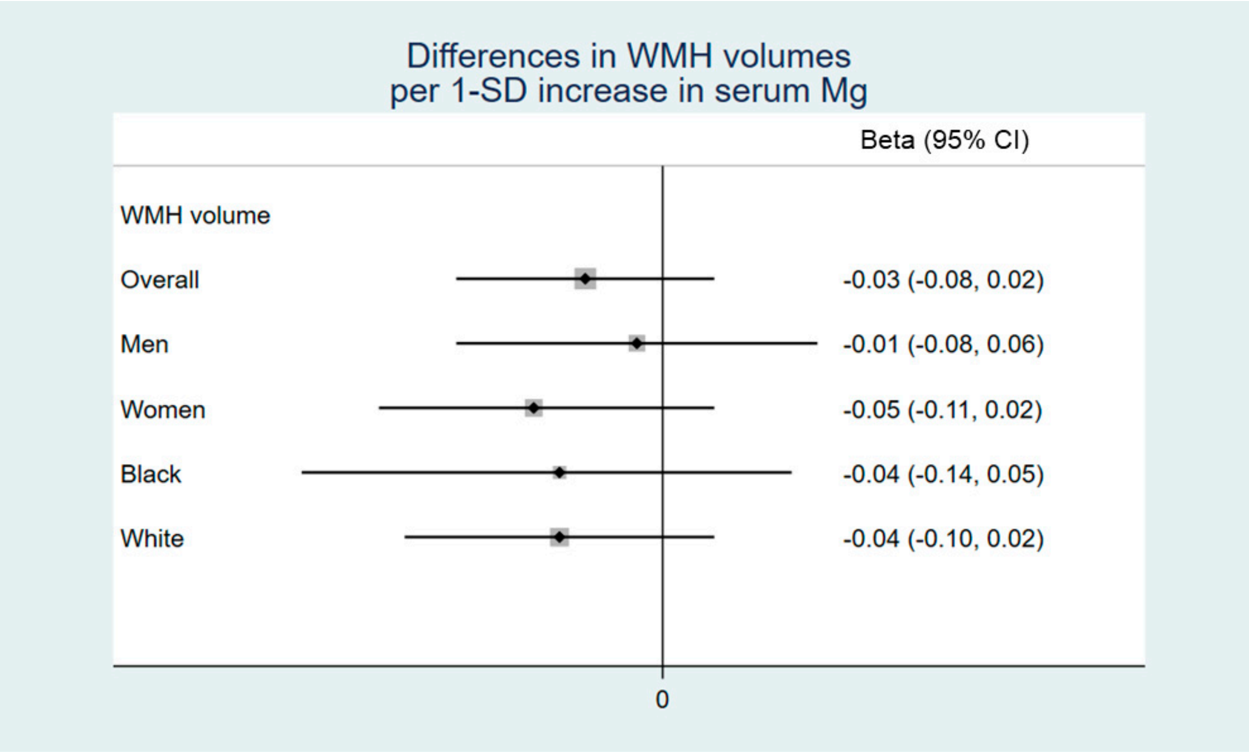

*Multiple linear regression models adjusted for age, sex, race/center, education, total intracranial volume, LDL and HDL cholesterol, body-mass index, sodium, potassium, calcium, smoking status, hypertension, hypertension medication use, history of coronary heart disease and heart failure, diabetes, eGFR, c-reactive protein, APOE allele. 1-SD Mg: 0.2 mg/dL.

Supplemental Table S1. Associations of serum magnesium with brain volumes, by sex, ARIC-NCS 2011–2013.

	Q1	Q2	Q3	Q4	Q5	1-SD Mg
**Male = 585**						
Total brain volume						
Model 1*	Ref.	0.19 (0.02, 0.36)	0.23 (0.07, 0.39)	0.21 (0.06, 0.36)	0.33 (0.17, 0.49)	0.10 (0.04, 0.15)
Model 2**	Ref.	0.16 (-0.01, 0.33)	0.19 (0.04, 0.35)	0.18 (0.03, 0.33)	0.28 (0.12, 0.44)	0.08 (0.02, 0.13)
Frontal lobe						
Model 1	Ref.	0.15 (-0.06, 0.37)	0.25 (0.05, 0.45)	0.18 (-0.03, 0.38)	0.32 (0.12, 0.52)	0.09 (0.02, 0.15)
Model 2	Ref.	0.15 (-0.06, 0.35)	0.23 (0.03, 0.43)	0.17 (-0.03, 0.37)	0.30 (0.10, 0.50)	0.08 (0.01, 0.15)
Temporal lobe						
Model 1	Ref.	0.29 (0.10, 0.48)	0.15 (-0.05, 0.34)	0.17 (-0.01, 0.36)	0.32 (0.12, 0.52)	0.10 (0.03, 0.16)
Model 2	Ref.	0.28 (0.09, 0.48)	0.15 (-0.04, 0.35)	0.20 (-0.02, 0.37)	0.31 (0.10, 0.53)	0.09 (0.02, 0.16)
Occipital lobe						
Model 1	Ref.	0.27 (0.05, 0.48)	0.12 (-0.10, 0.35)	0.27 (0.06, 0.48)	0.30 (0.08, 0.52)	0.09 (0.01, 0.17)
Model 2	Ref.	0.21 (0.004, 0.42)	0.04 (-0.18, 0.26)	0.18 (-0.03, 0.40)	0.17 (-0.07, 0.41)	0.04 (-0.05, 0.13)

Parietal lobe						
Model 1	Ref.	0.36 (0.15, 0.56)	0.22 (0.02, 0.42)	0.29 (0.09, 0.49)	0.43 (0.22, 0.64)	0.12 (0.05, 0.19)
Model 2	Ref.	0.32 (0.11, 0.53)	0.19 (-0.01, 0.38)	0.27 (0.07, 0.46)	0.38 (0.17, 0.60)	0.10 (0.03, 0.17)
Deep grey matter						
Model 1	Ref.	0.08 (-0.13, 0.30)	0.17 (-0.03, 0.37)	0.21 (0.02, 0.40)	0.33 (0.13, 0.52)	0.13 (0.07, 0.19)
Model 2	Ref.	0.06 (-0.16, 0.28)	0.13 (-0.07, 0.34)	0.20 (0.00, 0.39)	0.30 (0.10, 0.50)	0.12 (0.05, 0.18)
**Female = 881**						
Total brain volume						
Model 1*	Ref.	0.07 (-0.03, 0.18)	0.19 (0.09, 0.29)	0.13 (0.02, 0.23)	0.15 (0.05, 0.26)	0.05 (0.02, 0.08)
Model 2**	Ref.	0.05 (-0.05, 0.15)	0.19 (0.09, 0.29)	0.12 (0.02, 0.23)	0.16 (0.05, 0.27)	0.05 (0.02, 0.09)
Frontal lobe						
Model 1	Ref.	0.08 (-0.05, 0.21)	0.24 (0.12, 0.36)	0.10 (-0.02, 0.22)	0.14 (0.01, 0.27)	0.04 (0.00, 0.08)
Model 2	Ref.	0.04 (-0.10, 0.17)	0.20 (0.07, 0.32)	0.04 (-0.08, 0.17)	0.09 (-0.04, 0.22)	0.03 (-0.01, 0.07)
Temporal lobe						
Model 1	Ref.	0.03 (-0.11, 0.17)	0.11 (-0.02, 0.23)	0.07 (-0.05, 0.19)	0.07 (-0.05, 0.19)	0.04 (0.00, 0.08)
Model 2	Ref.	0.01 (-0.13, 0.15)	0.10 (-0.02, 0.23)	0.06 (-0.06, 0.18)	0.08 (-0.04, 0.19)	0.04 (0.01, 0.08)
Occipital lobe						
Model 1	Ref.	0.08 (-0.10, 0.26)	0.21 (0.06, 0.36)	0.09 (-0.05, 0.24)	0.06 (-0.08, 0.20)	0.04 (-0.01, 0.09)
Model 2	Ref.	0.06 (-0.11, 0.24)	0.18 (0.03, 0.33)	0.06 (-0.09, 0.21)	0.02 (-0.12, 0.17)	0.02 (-0.03, 0.07)
Parietal lobe						
Model 1	Ref.	0.05 (-0.08, 0.18)	0.18 (0.05, 0.31)	0.07 (-0.05, 0.18)	0.07 (-0.05, 0.19)	0.04 (0.01, 0.07)
Model 2	Ref.	0.04 (-0.09, 0.17)	0.18 (0.05, 0.31)	0.05 (-0.07, 0.18)	0.06 (-0.07, 0.19)	0.04 (0.00, 0.08)
Deep grey matter						
Model 1	Ref.	0.11 (-0.05, 0.28)	0.07 (-0.07, 0.21)	0.09 (-0.06, 0.24)	0.04 (-0.13, 0.22)	0.01 (-0.05, 0.07)
Model 2	Ref.	0.09 (-0.07, 0.25)	0.07 (-0.07, 0.21)	0.09 (-0.07, 0.24)	0.06 (-0.11, 0.22)	0.02 (-0.05, 0.08)
**Sex-magnesium interaction**						
Total brain volume			P = 0.32			
Frontal lobe			P = 0.19			
Temporal lobe			P = 0.22			
Occipital lobe			P = 0.19			
Parietal lobe			P = 0.03			
Deep grey matter			P = 0.04			

*Model 1 results from multiple linear regression adjusted for age, race/center, education and total intracranial volume. 1-SD Mg: 0.2 mg/dL.

**Model 2 results from multiple linear regression adjusted for model 1, plus LDL and HDL cholesterol, body-mass index, sodium, potassium, calcium, smoking status, hypertension, hypertension medication use, history of coronary heart disease and heart failure, diabetes, eGFR, c-reactive protein, APOE allele. 1-SD Mg: 0.2 mg/Dl.

Supplemental Table S2. Associations of serum magnesium with brain volumes, by race, ARIC-NCS 2011–2013.

	Q1	Q2	Q3	Q4	Q5	1-SD Mg
**Black = 422**						
Total brain volume						
Model 1*	Ref.	0.13 (0.01, 0.25)	0.21 (0.09, 0.33)	0.11 (-0.01, 0.23)	0.12 (-0.02, 0.26)	0.04 (0.00, 0.08)
Model 2**	Ref.	0.11 (-0.01, 0.23)	0.20 (0.08, 0.32)	0.09 (-0.04, 0.22)	0.14 (0.00, 0.27)	0.04 (0.01, 0.08)
Frontal lobe						
Model 1	Ref.	0.12 (-0.03, 0.27)	0.35 (0.20, 0.51)	0.26 (0.10, 0.42)	0.23 (0.05, 0.42)	0.09 (0.04, 0.14)
Model 2	Ref.	0.10 (-0.06, 0.25)	0.34 (0.18, 0.50)	0.25 (0.09, 0.41)	0.27 (0.07, 0.46)	0.10 (0.05, 0.15)
Temporal lobe						
Model 1	Ref.	0.06 (-0.11, 0.22)	0.12 (-0.06, 0.29)	-0.02 (-0.18, 0.15)	0.02 (-0.18, 0.22)	0.02 (-0.04, 0.07)
Model 2	Ref.	0.06 (-0.11, 0.22)	0.12 (-0.05, 0.30)	-0.03 (-0.20, 0.15)	0.07 (-0.15, 0.28)	0.03 (-0.03, 0.09)
Occipital lobe						
Model 1	Ref.	0.13 (-0.09, 0.34)	0.33 (0.13, 0.53)	0.27 (0.06, 0.47)	0.06 (-0.13, 0.25)	0.06 (0.00, 0.12)
Model 2	Ref.	0.10 (-0.11, 0.31)	0.27 (0.07, 0.47)	0.23 (0.02, 0.44)	0.10 (-0.10, 0.29)	0.06 (0.01, 0.12)
Parietal lobe						
Model 1	Ref.	0.14 (-0.02, 0.29)	0.30 (0.13, 0.46)	0.07 (-0.11, 0.24)	0.12 (-0.06, 0.30)	0.05 (0.00, 0.10)
Model 2	Ref.	0.13 (-0.02, 0.29)	0.28 (0.13, 0.44)	0.05 (-0.12, 0.23)	0.14 (-0.05, 0.33)	0.06 (0.01, 0.10)
Deep grey matter						
Model 1	Ref.	0.10 (-0.13, 0.33)	0.07 (-0.11, 0.24)	0.03 (-0.17, 0.23)	0.12 (-0.10, 0.34)	0.02 (-0.04, 0.08)
Model 2	Ref.	0.08 (-0.14, 0.30)	0.08 (-0.10, 0.26)	0.05 (-0.16, 0.27)	0.16 (-0.07, 0.39)	0.04 (-0.03, 0.11)
**White = 1,044**						
Total brain volume						
Model 1*	Ref.	0.09 (-0.02, 0.22)	0.19 (0.07, 0.30)	0.16 (0.05, 0.26)	0.23 (0.12, 0.34)	0.07 (0.04, 0.11)
Model 2**	Ref.	0.07 (-0.04, 0.19)	0.16 (0.05, 0.27)	0.13 (0.03, 0.24)	0.19 (0.08, 0.31)	0.06 (0.02, 0.10)
Frontal lobe						
Model 1	Ref.	0.06 (-0.09, 0.21)	0.16 (0.03, 0.29)	0.06 (-0.07, 0.19)	0.16 (0.03, 0.28)	0.05 (0.00, 0.09)
Model 2	Ref.	0.06 (-0.08, 0.21)	0.14 (0.003, 0.27)	0.02 (-0.11, 0.15)	0.11 (-0.02, 0.24)	0.02 (-0.02, 0.07)
Temporal lobe						
Model 1	Ref.	0.15 (0.01, 0.29)	0.10 (-0.03, 0.23)	0.14 (0.02, 0.26)	0.19 (0.07, 0.31)	0.07 (0.03, 0.11)
Model 2	Ref.	0.14 (0.00, 0.28)	0.09 (-0.04, 0.22)	0.12 (0.00, 0.25)	0.16 (0.03, 0.28)	0.06 (0.02, 0.11)
Occipital lobe						
Model 1	Ref.	0.14 (-0.03, 0.31)	0.09 (-0.07, 0.25)	0.11 (-0.04, 0.26)	0.13 (-0.01, 0.28)	0.05 (-0.01, 0.10)
Model 2	Ref.	0.11 (-0.06, 0.28)	0.03 (-0.13, 0.19)	0.04 (-0.11, 0.19)	0.05 (-0.11, 0.20)	0.02 (-0.04, 0.07)
Parietal lobe						
Model 1	Ref.	0.15 (0.01, 0.29)	0.11 (-0.03, 0.25)	0.15 (0.02, 0.27)	0.19 (0.06, 0.32)	0.07 (0.03, 0.11)
Model 2	Ref.	0.14 (-0.01, 0.28)	0.09 (-0.04, 0.23)	0.13 (0.00, 0.26)	0.16 (0.02, 0.30)	0.06 (0.02, 0.11)
Deep grey matter						
Model 1	Ref.	0.08 (-0.08, 0.24)	0.09 (-0.05, 0.24)	0.14 (0.00, 0.28)	0.15 (0.00, 0.31)	0.06 (-0.01, 0.12)

Model 2	Ref.	0.04 (-0.12, 0.20)	0.05 (-0.10, 0.19)	0.10 (-0.04, 0.25)	0.13 (-0.03, 0.28)	0.05 (-0.02, 0.11)
**Race-magnesium interaction**						
Total brain volume			P = 0.58			
Frontal lobe			P = 0.02			
Temporal lobe			P = 0.28			
Occipital lobe			P = 0.19			
Parietal lobe			P = 0.91			
Deep grey matter			P = 0.80			

*Model 1 results from multiple linear regression adjusted for age, sex, center, education and total intracranial volume. 1-SD Mg: 0.2 mg/dL.

**Model 2 results from multiple linear regression adjusted for model 1, plus LDL and HDL cholesterol, body-mass index, sodium, potassium, calcium, smoking status, hypertension, hypertension medication use, history of coronary heart disease and heart failure, diabetes, eGFR, c-reactive protein, APOE allele. 1-SD Mg: 0.2 mg/dL.

Supplemental Table S3. Associations of serum magnesium with subclinical cerebrovascular disease, by sex, ARIC-NCS 2011–2013.

Variable	Q1	Q2	Q3	Q4	Q5	1-SD Mg
**Male = 585**						
	Odds Ratios (95%CI)					
Cortical infarcts						
Model 1*	1 (ref.)	0.88 (0.33, 2.37)	0.71 (0.27, 1.86)	0.67 (0.28, 1.59)	0.67 (0.24, 1.88)	0.91 (0.66, 1.25)
Model 2**	1 (ref.)	1.04 (0.37, 2.91)	0.78 (0.25, 2.42)	0.84 (0.30, 2.31)	0.72 (0.22, 2.32)	0.94 (0.63, 1.40)
Subcortical infarcts						
Model 1	1 (ref.)	0.85 (0.38, 1.90)	0.58 (0.27, 1.24)	0.46 (0.23, 0.93)	0.47 (0.21, 1.02)	0.75 (0.59, 0.95)
Model 2	1 (ref.)	0.94 (0.39, 2.28)	0.63 (0.26, 1.57)	0.52 (0.23, 1.17)	0.48 (0.19, 1.24)	0.75 (0.55, 1.01)
Lacunar infarcts						
Model 1	1 (ref.)	0.82 (0.37, 1.85)	0.58 (0.27, 1.24)	0.46 (0.23, 0.93)	0.42 (0.19, 0.93)	0.73 (0.57, 0.93)
Model 2	1 (ref.)	0.90 (0.37, 2.20)	0.62 (0.25, 1.54)	0.51 (0.22, 1.14)	0.43 (0.16, 1.11)	0.73 (0.54, 0.99)
	Beta (95%CI)					
Ln(WMH volume)†						
Model 1	Ref.	-0.12 (-0.34, 0.10)	0.04 (-0.18, 0.27)	-0.06 (-0.30, 0.17)	-0.12 (-0.34, 0.11)	-0.02 (-0.09, 0.05)
Model 2	Ref.	-0.11 (-0.33, 0.11)	0.05 (-0.17, 0.27)	-0.04 (-0.28, 0.20)	-0.08 (-0.31, 0.14)	-0.01 (-0.08, 0.06)
**Female = 881**						
	Odds Ratios (95%CI)					
Cortical infarcts						
Model 1	1 (ref.)	1.37 (0.53, 3.52)	1.50 (0.63, 3.59)	1.32 (0.56, 3.11)	1.02 (0.42, 2.51)	1.11 (0.86, 1.44)
Model 2	1 (ref.)	1.16 (0.43, 3.12)	1.50 (0.61, 3.71)	1.54 (0.61, 3.91)	1.12 (0.43, 2.90)	1.21 (0.91, 1.59)

Subcortical infarcts						
Model 1	1 (ref.)	0.87 (0.43, 1.77)	0.38 (0.19, 0.75)	0.63 (0.33, 1.22)	0.36 (0.18, 0.71)	0.75 (0.62, 0.91)
Model 2	1 (ref.)	0.96 (0.47, 1.97)	0.39 (0.19, 0.81)	0.63 (0.32, 1.23)	0.34 (0.17, 0.70)	0.75 (0.61, 0.92)
Lacunar infarcts						
Model 1	1 (ref.)	0.87 (0.43, 1.76)	0.38 (0.19, 0.76)	0.62 (0.32, 1.20)	0.33 (0.16, 0.67)	0.74 (0.61, 0.90)
Model 2	1 (ref.)	0.95 (0.46, 1.97)	0.40 (0.19, 0.82)	0.61 (0.30, 1.20)	0.31 (0.15, 0.65)	0.73 (0.60, 0.90)
	Beta (95%CI)					
Ln(WMH volume)†						
Model 1	Ref.	0.11 (-0.08, 0.30)	-0.27 (-0.45, -0.09)	-0.23 (-0.44, -0.02)	-0.11 (-0.29, 0.08)	-0.07 (-0.13, -0.01)
Model 2	Ref.	0.15 (-0.05, 0.35)	-0.23 (-0.41, -0.04)	-0.17 (-0.38, 0.04)	-0.07 (-0.25, 0.12)	-0.05 (-0.11, 0.02)
**Sex-magnesium interaction**						
Cortical infarcts			P = 0.41			
Subcortical infarcts			P = 0.74			
Lacunar infarcts			P = 0.77			
Ln(WMH volume)			P = 0.65			

Results from logistic regression (infarcts) and linear regression (WMH volume) adjusted for:
*Model 1: age, race/center, and education. 1-SD Mg: 0.2 mg/dL.
**Model 2: model 1, plus LDL and HDL cholesterol, body-mass index, sodium, potassium, calcium, smoking status, hypertension, hypertension medication use, history of coronary heart disease and heart failure, diabetes, eGFR, c-reactive protein, APOE allele. 1-SD Mg: 0.2 mg/dL.
† Additionally adjusted for total intracranial volume. WMH: white matter hyperintensities.

Supplemental Table S4. Associations of serum magnesium with subclinical cerebrovascular disease, by race, ARIC-NCS 2011-2013.

Variable	Q1	Q2	Q3	Q4	Q5	1-SD Mg
**Black = 422**						
	Odds Ratios (95%CI)					
Cortical infarcts						
Model 1*	1 (ref.)	0.48 (0.15, 1.51)	0.62 (0.24, 1.59)	0.34 (0.13, 0.91)	0.53 (0.20, 1.40)	0.95 (0.68, 1.32)
Model 2**	1 (ref.)	0.68 (0.19, 2.38)	0.80 (0.27, 2.34)	0.56 (0.18, 1.75)	0.86 (0.28, 2.62)	1.16 (0.77, 1.74)
Subcortical infarcts						
Model 1	1 (ref.)	0.67 (0.32, 1.40)	0.78 (0.38, 1.62)	0.31 (0.13, 0.75)	0.57 (0.22, 1.45)	0.78 (0.60, 1.02)
Model 2	1 (ref.)	0.78 (0.35, 1.74)	0.88 (0.41, 1.90)	0.34 (0.13, 0.93)	0.56 (0.19, 1.67)	0.79 (0.59,1.05)
Lacunar infarcts						
Model 1	1 (ref.)	0.62 (0.29, 1.31)	0.82 (0.39, 1.71)	0.32 (0.13, 0.79)	0.60 (0.23, 1.52)	0.80 (0.61, 1.04)
Model 2	1 (ref.)	0.71 (0.31, 1.61)	0.91 (0.42, 1.98)	0.36 (0.13, 0.98)	0.60 (0.20, 1.77)	0.81 (0.60, 1.08)
	Beta (95%CI)					

Ln(WMH volume)†						
Model 1	Ref.	-0.09 (-0.33, 0.16)	-0.31 (-0.54, -0.08)	-0.27 (-0.49, -0.05)	-0.08 (-0.37, 0.21)	-0.07 (-0.16, 0.03)
Model 2	Ref.	-0.07 (-0.32, 0.18)	-0.26 (-0.49, -0.03)	-0.17 (-0.40, 0.07)	-0.05 (-0.34, 0.24)	-0.04 (-0.14, 0.05)
**White = 1,044**						
	Odds Ratios (95%CI)					
Cortical infarcts						
Model 1	1 (ref.)	1.93 (0.78, 4.75)	1.67 (0.69, 4.08)	1.65 (0.71, 3.83)	1.18 (0.48, 2.88)	1.04 (0.82, 1.31)
Model 2	1 (ref.)	1.95 (0.75, 5.08)	1.76 (0.69, 4.48)	1.79 (0.74, 4.33)	1.24 (0.47, 3.28)	1.16 (0.77, 1.74)
Subcortical infarcts						
Model 1	1 (ref.)	0.92 (0.47, 1.79)	0.38 (0.20, 0.74)	0.18 (0.33, 1.10)	0.12 (0.20, 0.71)	0.74 (0.61, 0.88)
Model 2	1 (ref.)	0.96 (0.48, 1.91)	0.41 (0.20, 0.84)	0.61 (0.33, 1.13)	0.40 (0.20, 0.79)	0.76 (0.62, 0.93)
Lacunar infarcts						
Model 1	1 (ref.)	0.92 (0.47, 1.80)	0.38 (0.20, 0.74)	0.59 (0.33, 1.08)	0.34 (0.18, 0.64)	0.72 (0.60, 0.86)
Model 2	1 (ref.)	0.97 (0.49, 1.93)	0.41 (0.20, 0.84)	0.60 (0.32, 1.12)	0.36 (0.18, 0.72)	0.74 (0.61, 0.91)
	Beta (95%CI)					
Ln(WMH volume)†						
Model 1	Ref.	0.01 (-0.17, 0.19)	-0.15 (-0.32, 0.02)	-0.16 (-0.36, 0.04)	-0.14 (-0.31, 0.02)	-0.06 (-0.12, -0.01)
Model 2	Ref.	0.01 (-0.18, 0.20)	-0.11 (-0.29, 0.07)	-0.12 (-0.32, 0.07)	-0.09 (-0.27, 0.08)	-0.04 (-0.10, 0.02)
**Race-magnesium interaction**						
Cortical infarcts			P = 0.24			
Subcortical infarcts			P = 0.89			
Lacunar infarcts			P = 0.84			
Ln(WMH volume)			P = 0.70			

Results from logistic regression (infarcts) and linear regression (WMH volume) adjusted for:
*Model 1: age, sex, center, and education. 1-SD Mg: 0.2 mg/dL.
**Model 2: model 1, plus LDL and HDL cholesterol, body-mass index, sodium, potassium, calcium, smoking status, hypertension, hypertension medication use, history of coronary heart disease and heart failure, diabetes, eGFR, c-reactive protein, APOE allele. 1-SD Mg: 0.2 mg/dL.
† Additionally adjusted for total intracranial volume. WMH: white matter hyperintensities.
